# No beneficial effect on survival but a decrease in postoperative complications in patients with rectal cancer undergoing robotic surgery: a retrospective cohort study

**DOI:** 10.1186/s12893-021-01309-w

**Published:** 2021-09-28

**Authors:** Xiong Lei, Lingling Yang, Zhixiang Huang, Haoran Shi, Zhen Zhou, Cheng Tang, Taiyuan Li

**Affiliations:** 1 Department of General Surgery, The First Affiliated Hospital of Nanchang University, Nanchang, 330006 Jiangxi China; 2 Gastrointernal Surgical Institute, Nanchang University, Nanchang, 330006 Jiangxi China; 3 Department of Gastroenterology, The Second Affiliated Hospital of Nanchang University, Nanchang, 330006 Jiangxi China

**Keywords:** Robotic surgery, Laparoscopic surgery, Rectal cancer, Oncologic outcome

## Abstract

**Background:**

Robotic surgery has been taken as a new modality to surpass the technical limitations of conventional surgery. Here we aim to compare the oncologic outcomes of patients with rectal cancer receiving robotic vs. laparoscopic surgery.

**Methods:**

Data from patients diagnosed with rectal cancer between March 2011 and December 2018 were obtained for outcome assessment at the First Affiliated Hospital of Nanchang University. All patients were separated into two groups: a robot group (patients receiving robotic surgery, n = 314) and a laparoscopy group (patients receiving laparoscopic surgery, n = 220). The primary endpoint was survival outcomes. The secondary endpoints were the general conditions of the operation, postoperative complications and pathological characteristics.

**Results:**

The 5-year overall survival (OS) and disease-free survival (DFS) at years 1, 3 and 5 were 96.6%, 88.7%, and 87.7% vs. 96.7%, 88.1%, and 78.4%, and 98.6%, 80.2-, and 73.5% vs. 96.2-, 87.2-, and 81.1% in the robot and laparoscopy groups, respectively (*P* > 0.05). In the multivariable-adjusted analysis, robotic surgery was not an independent prognostic factor for OS and DFS (P = 0.925 and 0.451, respectively). With respect to the general conditions of the operation, patients in the robot group had significantly shorter operation times (163.5 ± 40.9 vs. 190.5 ± 51.9 min), shorter times to 1^st^ gas passing [2(1) vs. 3(1)d] and shorter hospital stay days [7(2) vs. 8(3)d] compared to those in the laparoscopy group (*P* < 0.01, respectively). After the operation, the incidence of short- and long-term complications in the robot group was significantly lower than that in the laparoscopy group (15.9% vs. 32.3%; *P* < 0.001), especially for urinary retention (1.9% vs. 7.3%; 0.6% vs. 4.1%, *P* < 0.05, respectively). With regard to pathological characteristics, TNM stages II and III were more frequently observed in the robot group than in the laparoscopy group (94.3% vs. 83.2%, *P* < 0.001). No significant difference were observed in lymph nodes retrieved, lymphovascular invasion and circumferential resection margin involvement between the two groups (*P* > 0.05, respectively).

**Conclusions:**

This monocentre retrospective comparative cohort study revealed short-term advantages of robot-assisted rectal cancer resection but similar survival compared to conventional laparoscopy.

## Background

Colorectal cancer ranks third in terms of cancer incidence but second in terms of mortality worldwide [1]. Thus, there is an urgent need to improve every treatment method for colorectal cancer, including surgical procedures. Minimally invasive techniques have allowed the use of laparoscopic approaches in the treatment of patients with colorectal cancer based on similar or better perioperative and oncologic outcomes [2, 3], and they have been regarded as an alternative to conventional open surgery [4–6]. However, in rectal cancer, a laparoscopic approach is quite different and more difficult than that in colon cancer. Procedures such as dissection deep into the pelvis to accomplish total mesorectal excision (TME) and to obtain a specimen with complete margins, as well as a safe anastomosis, are technically challenging. Surgeons are faced with challenges such as a narrow pelvic cavity, anatomical complexity, and restricted surgical view during laparoscopic surgery, although previous studies reported that laparoscopic rectal cancer surgery was feasible [7, 8]. However, laparoscopic rectal surgery has been associated with limited dexterity with nonarticulating unstable instruments, unnatural hand–eye coordination, and flat 2-dimensional (2D) vision [9]. Thus, the robotic system seems potentially suited for the surgical treatment of rectal cancer because of its theoretical advantages, and it has been introduced in many centres since its first adoption in 2001 [10].

The current robotic surgical system provides advanced technology and has advantages in rectal cancer resection. Several previous studies documented that robotic surgery is equivalent to laparoscopic surgery with respect to perioperative and oncologic outcomes [11–13]. To date, few reports with data have evaluated the short-term and long-term outcomes of robotic surgery compared with laparoscopic surgery for rectal cancer. No sufficient data from a large cohort are available to support the adoption of the robotic system for rectal cancer instead of laparoscopic surgery.

Our centre is one of the earliest hospitals to introduce the da Vinci^®^ surgical system (Intuitive Surgical, Sunnyvale, CA, USA) in China. Here, We evaluate the oncologic outcomes of rectal cancer by robotic surgery or conventional laparoscopic surgery including the primary endpoint of survival outcomes and the secondary endpoints of the general conditions of the operation, postoperative complications and pathological characteristics.

## Methods

### Patient selection

The prospectively collected records of all patients at the First Affiliated Hospital of Nanchang University between March 2011 and December 2018 with histologically proven rectal adenocarcinoma were retrospectively reviewed. All patients were separated into two groups: a robot group in which the patients received robotic surgery, and a laparoscopy group in which the patients received laparoscopic surgery. All patients included in this study met the following criteria: (1) the disease was histologically defined rectal adenocarcinoma; (2) all the patients underwent TME; (3) tumour size was measurable, and pathological evaluation records of pelvic lymph nodes were complete; (4) the patient had no history of malignancy in other organs; and (5) the clinicopathological and follow-up data of the patients were complete. The exclusion criteria were as follows: (1) age > 80 and < 18 y; (2) other malignant tumours; (3) TNM stage at 0, IV; (4) multivisceral resection; (5) palliative resection; (6) restaging surgery; (7) abdominal and pelvic exploration only; and (8) incomplete patient information.

Before surgery, all patients were informed of the detailed characteristics of both robotic and laparoscopic surgical procedures. After informed consent was obtained, the patients decided their preferred approach. The study protocol followed the Ethical Guidelines of the 1975 Declaration of Helsinki, revised in 2000. All related procedures were performed with the approval of the Internal Review and the Ethics Boards of the First Affiliated Hospital, Nanchang University.

### Data collection

The following clinical and demographic information was collected: age, sex, body mass index (BMI), carcinoembryonic antigen (CEA), American Society of Anesthesiologists (ASA) class, tumour location from the anal verge, preoperative chemoradiotherapy (CRT), and clinical T stage. The intraoperative and perioperative conditions (e.g., operation time, intraoperative bleeding, and complications), postoperative complications (e.g., anastomotic leakage, bleeding, wound problems, urinary retention, and the development of an ileus) and survival time were also collected. The baseline characteristics of patients at enrolment were assessed within 24 h before robotic or laparoscopic surgery.

### Outcome evaluation

The primary endpoint for this study was survival outcomes, including overall survival (OS) and disease-free survival (DFS). The secondary endpoints were the general conditions of operation, postoperative complications and pathologic characteristics. The general conditions of the operation included operation time, bleeding volume, time to 1st gas passing and length of hospital stay. The postoperative complications associated with robotic and laparoscopic surgery included short- and long-term complications, which were defined as complications that occurred less than 30 days or more than 30 days after the operation, respectively [14]. The recorded long-term complications were ileus, urinary retention, adhesions, incisional hernias, anastomotic strictures and rectovaginal/rectovesical fistulas. The pathologic characteristics included TNM stage, differentiation grade, lymph nodes retrieved, lymphovascular invasion and circumferential resection margin (CRM) involvement.

Patients were followed up with for 5 years or until Dec 31, 2018. Operative death was defined as death within 30 days of the operation or any time after the operation if the patient did not leave the hospital alive.

### Surgical procedures

In our retrospective study, patients who underwent elective laparoscopic or robotic surgery for stages I–III rectal cancer between March 2011 and December 2018 were included. Patients who underwent palliative surgery, intersphincteric resection, abdominoperineal resection, or lateral pelvic lymph node dissection or patients who had hereditary colorectal cancer or distant metastasis were excluded. Since the robotic system was introduced in March 2016, selection of the surgical approach was determined after discussion with patients about the differences and higher costs of robotic surgery. Before March 2016, patients were chosen to undergo laparoscopic surgery. All procedures, including robotic surgery using the da Vinci^®^ surgical system and laparoscopic surgery, were performed or supervised by a single surgeon (T-Y Li). Briefly, the same principle and steps were applied in both the laparoscopic and robotic surgery procedures: ligature of inferior mesenteric blood vessels close to the origin, mobilization of the sigmoid colon and rectum using sharp dissection, complete splenic flexure takedown for mid- and low rectal cancer, clamping below the tumour, and washing of the rectal stump with 10% povidone-iodine before rectal transection. End-to-end anastomosis was then performed by either mechanical circular stapling or manual anastomosis. The anastomosis was tested with air inflation. Abdominoperineal excision was performed when the levator ani muscle had been invaded by the tumour. In some patients, a temporary ileostomy was conducted to protect the anastomosis, with digestive tract reconstruction performed 3 months later.

### Statistical analysis

In the univariate statistical analyses, the χ^2^ test or Fisher’s exact test was used for categorical variables. Student’s t-test and the Mann–Whitney U test were used for continuous variables. The results are presented as the frequency (percentage), mean ± standard deviation (SD) or median (interquartile range). Survival curves were obtained by the Kaplan–Meier method, and the OS and DFS rates were compared by the log-rank test. The Cox proportional hazard regression model was used to identify factors that were independently associated with OS and DFS. The candidate covariates for univariate analysis included age, sex, BMI, CEA, ASA class, tumour location, robotic surgery, preoperative CRT, previous abdominal surgery, lymph nodes retrieved, proximal resection margin (PRM), distal resection margin (DRM), TNM stage, differentiation grade, circumferential resection margin (CRM), and lymphovascular invasion. Only factors with a *P* < 0.05 in the univariate analysis could be included in the multivariate analysis using a stepwise method, and variables with a *P* < 0.05 and hazard ratio (HR) > 20% were kept in the final model. A two-tailed *P* < 0.05 was considered significant. All statistical analyses were performed using the SPSS 22 software package (SPSS, Chicago, Illinois, USA).

## Results

### Patients and clinical characteristics in the robot and laparoscopy groups

**Fig. 1:**
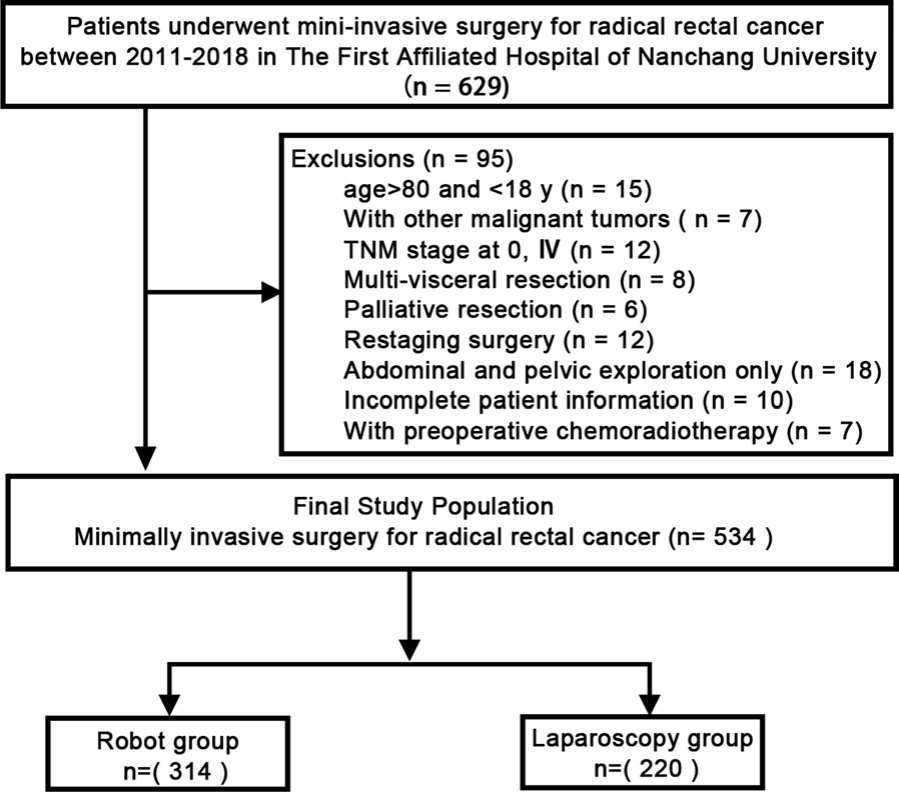
Flowchart of patient selection. Robot group, patients who underwent robotic surgery; Laparoscopy group, patients who underwent laparoscopic surgery

A total of 629 patients were initially screened, and 534 patients with rectal adenocarcinoma were finally enrolled in this study; 314 patients were in the robot group, and 220 patients were in the laparoscopy group (Fig. 1). The clinical characteristics of the two groups of patients are presented in Table 1. A similar sex distribution was observed in the robot and laparoscopy groups, and most patients were men. Age, BMI, preoperative serum CEA, ASA class and previous abdominal surgery were not significantly different between the two groups. Tumour location from the anal verge was significantly shorter in the robot group than in the laparoscopy group (5.9 ± 2.6 cm vs. 8.5 ± 3.6 cm, *P* < 0.001).

**Table 1:** Clinical and pathological characteristics of patients with rectal adenocarcinoma during hospitalization at enrolment

	Robot (n = 314)	Laparoscopy (n = 220)	*P* value
Age	58.9 ± 12.4	58.8 ± 12.4	0.989
Male, no. (%)	194 (61.9%)	146 (66.4%)	0.264
BMI (kg/m^2^)	22.5 ± 3.0	22.3 ± 2.9	0.901
CEA	9.3 ± 27.3	8.7 ± 16.4	0.784
ASA class, no. (%)			
1	174 (55.4%)	119 (54.0%)	0.224
2	128 (40.7%)	92 (41.5%)	
3	12 (3.8%)	9 (4.1%)	
Tumour location from the anal verge (cm)	5.9 ± 2.6	8.5 ± 3.6	** < 0.001**
With previous abdominal surgery	30 (9.5%)	25 (11.2%)	0.520

Data are expressed as the mean ± standard deviation (SD) or number of patients (percentage). The continuous variables were compared by using Student’s t-test and the Mann–Whitney U test, and the categorical variables were compared by using the χ^2^ test or Fisher’s exact test between the Robot and Laparoscopy groups

Bold values indicate statistical significance

*Defined by magnetic-resonance imaging (MRI)

*BMI* body mass index, *CEA* carcinoembryonic antigen, *ASA* American Society of Anesthesiologists, *CRT* chemoradiotherapy, *CRM* circumferential resection margin

### Survival analyses and prognostic factors

**Fig. 2:**
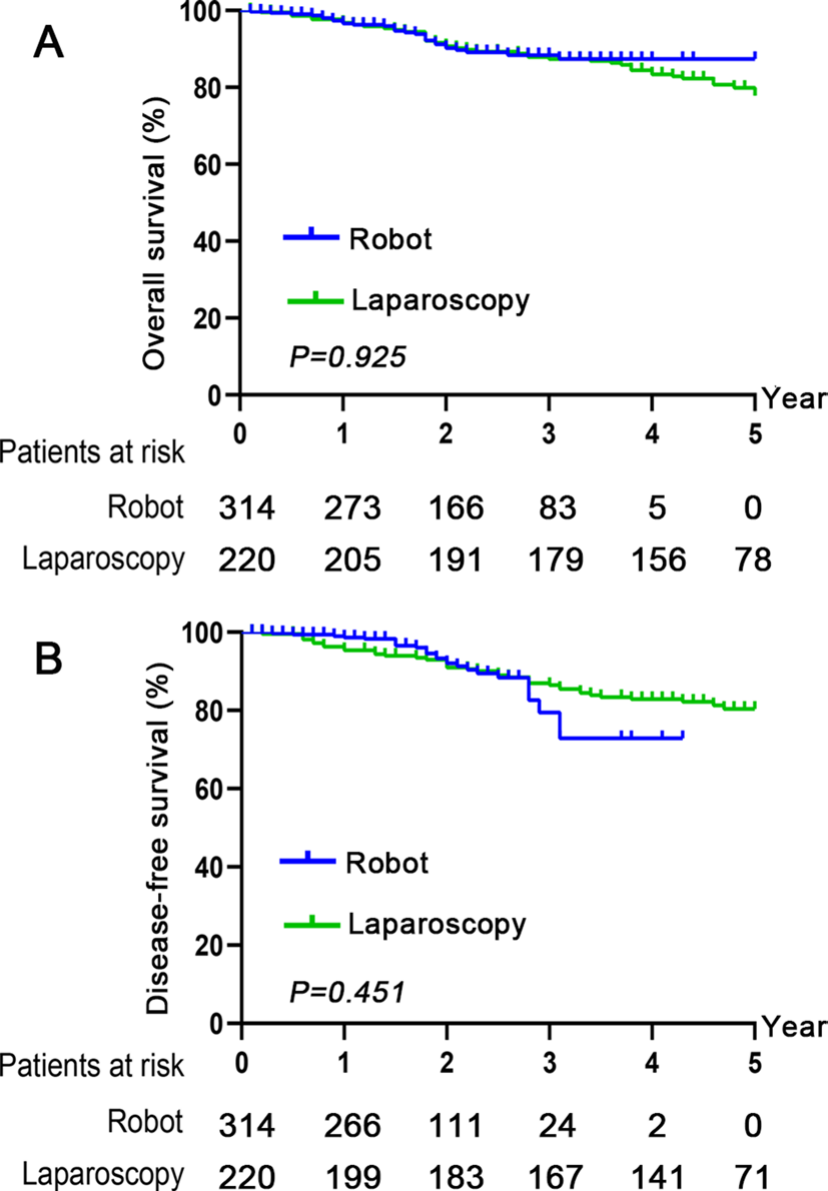
Kaplan–Meier survival curves for OS and DFS in the robot and laparoscopy groups. **A** The 1-, 3- and 5-year OS rates were 96.6%, 88.7%, and 87.7% vs. 96.7%, 88.1%, and 78.4% in the robot and laparoscopy groups. **B** The 1-, 3- and 5-year DFS rates were 98.6%, 80.2%, and 73.5% vs. 96.2%, 87.2%, and 81.1% between the two groups. Both OS and DFS were not significantly different between the robot and laparoscopy groups (P > 0.05). OS, overall survival; DFS, disease-free survival

The OS rates at years 1, 3 and 5 in the robot and laparoscopy groups were 96.6%, 88.7%, and 87.7% vs. 96.7%, 88.1%, and 78.4%, respectively (*P* = 0.925, Fig. 2A). The DFS at years 1, 3 and 5 in the robot and laparoscopy groups was 98.6%, 80.2-, and 73.5% vs. 96.2-, 87.2-, and 81.1% (*P* = 0.451, Fig. 2B). No significant differences were observed between the two groups. In the univariate analysis, the factors associated with 5-year OS and DFS were age, CEA, tumour location, TNM stage, differentiation grade and lymphovascular invasion (Table 2). After adjusting for independent prognostic variables, TNM stage and differentiation grade were independent prognostic indicators for 5-year OS and DFS [HR (CI): 1.622 (1.068–2.464) and 1.919 (1.241–2.969) for OS and 1.664 (1.093–2.535) and 1.660 (1.116–2.469) for DFS, *P* < 0.05, respectively, Table 2]. The surgical approach (robot or laparoscopy) was not associated with significantly higher OS or DFS (*P* = 0.925 and 0.451, respectively, Table 2). These findings indicated that robotic surgery did not improve long-term survival compared with laparoscopic surgery.

**Table 2:** Univariate and multivariate analyses of factors associated with 5-year OS and 5-year DFS

Variables	5-year OS			5-year DFS		
	Univariate	Multivariate	*P*	Univariate	Multivariate	*P*
	*P*	HR (95% CI)		*P*	HR (95% CI)	
Age	0.024	1.026 (1.005–1.047)	0.014	0.011	0.979 (0.959–0.999)	0.039
Sex (male)	0.369			0.066		
BMI (kg/m^2^)	0.307			0.326		
CEA	< 0.001	1.016 (1.007–1.026)	0.001	0.058		
ASA class	0.053		0.669	0.032		
Tumour location	0.036		0.054	0.001	0.872 (0.802–0.948)	0.001
With robotic surgery	0.925			0.451		
With previous abdominal surgery	0.065			0.583		
Lymph nodes retrieved	0.332			0.036	0.937 (0.888–0.989)	0.018
TNM stage	**0.003**	1.622 (1.068–2.464)	**0.023**	**0.001**	1.919 (1.241–2.969)	**0.003**
Differentiation grade	**0.010**	1.664 (1.093–2.535)	**0.018**	**< 0.001**	1.660 (1.116–2.469)	**0.012**
CRM (no involved)	0.302			0.754		
With lymphovascular invasion	0.018		0.123	0.005		

Statistical analysis was performed using a multivariable Cox proportional hazards model. Significant variables from the univariate analysis were included in the multivariate Cox regression models to assess their contribution to 5-year OS and DFS. Variables with a P < 0.05 and hazard ratio (HR) > 20% were kept in the final model

Bold values indicate statistical significance

*BMI* body mass index, *ASA* American Society of Anesthesiologists, *CRT* chemoradiotherapy, *TNM* tumour node metastasis, *CRM* circumferential resection margin, *OS* overall survival, *DFS* disease-free survival, *HR* Hazard radio, *95% CI* 95% confidence interval

### The general conditions of the operation

The general conditions of the operation are listed in Table 3. The operation time was significantly decreased in the robot group compared with the laparoscopy group (163.5 ± 40.9 vs. 190.5 ± 51.9 min, *P* < 0.001). The time to 1st gas passing [2 (1) vs. 3 (1) d] and length of hospital stay [7 (2) vs. 8 (3) d] were significantly shorter in patients with robotic surgery than in those receiving laparoscopy surgery (*P* < 0.01, respectively). No significant differences were observed with regard to intraoperative bleeding volume or time to 1st soft diet or bowel movement.

**Table 3:** General conditions of operation

	Robot (n = 314)	Laparoscopy (n = 220)	*P* value
Intraoperative			
Operation time (min)	163.5 ± 40.9	190.5 ± 51.9	**< 0.001**
Conversion	0	0	
Bleeding (mL)	150 (100)	150 (100)	0.657
Adverse events			
Bladder injury	0	0	
Perforation of the rectum	0	0	
Disruption of colorectal anastomosis	0	0	
Postoperative			
Time to 1st gas passing (d)	2 (1)	3 (1)	**< 0.001**
Length of hospital stay (d)	7 (2)	8 (3)	**0.002**
Time to 1st soft diet (d)	4 (1)	4 (1)	0.784
Time to bowel movement (d)	2 (0)	2 (0)	0.553

Data are expressed as the mean ± standard deviation (SD), median (interquartile range) or number of patients (percentages). Continuous variables were compared by using Student’s t-test and the Mann–Whitney U test between the robot and laparoscopy groups

Bold values indicate statistical significance

### Postoperative complications

The type and proportion of postoperative complications associated with surgical treatment are described in Table 4. Postoperative complications occurred in 50 of the 314 patients in the robot group, which was significantly lower than that in the laparoscopy group, with 71 of the 220 patients (15.9% vs. 32.3%; *P* < 0.001). No significant differences were observed between the two groups with respect to the occurrence of short- and long-term complications, including anastomotic leakage, anastomotic bleeding, wound problems, ileus, intra-abdominal abscesses, anaemia, ascites, adhesions, incisional hernias, anastomotic strictures and rectovaginal/rectovesical fistulas (*P* > 0.05, respectively). However, the occurrence of short- and long-term urinary retention in the robot group was significantly lower than that in the laparoscopy group (1.9% vs. 7.3% and 0.6% vs. 4.1% *P* < 0.05, respectively). The above findings indicated that robotic surgery was associated with a decreased prevalence of postoperative complications.

**Table 4:** Short- and long-term postoperative complications

	Robot (n = 314)	Laparoscopy (n = 220)	*P* value
Total, no. (%)	50 (15.9%)	71 (32.3%)	**< 0.001**
Short-term			
Anastomotic leakage	16 (5.1%)	10 (4.5%)	0.771
Anastomotic bleeding	2 (0.6%)	2 (0.9%)	0.720
Wound problem	13 (4.1%)	6 (2.7%)	0.386
Urinary retention^*^	**6 (1.9%)**	**16(7.3%)**	**0.002**
Ileus	1 (0.3%)	0	0.402
Intra-abdominal abscess	4 (1.3%)	2 (0.9%)	0.694
Anaemia requiring transfusion	0	4 (1.8%)	0.059
Ascites	1 (0.3%)	1 (0.4%)	0.800
Long-term			
Ileus	2 (0.6%)	5 (2.3%)	0.212
** Urinary retention**^#^	**2 (0.6%)**	**9 (4.1%)**	**0.014**
Adhesions	0	2 (0.9%)	0.169
Incisional hernia	1 (0.3%)	3 (1.4%)	0.385
Anastomotic stricture	0	0	
Rectovaginal/rectovesical fistula	1 (0.3%)	1 (0.5%)	0.800

* Occurred in perioperative period but disappeared after effective therapy in one month; # still existed after one month' effective therapy; Data are expressed as the number of patients (percentage). Categorical variables were compared by using the χ^2^ test or Fisher’s exact test between the robot and laparoscopy groups

Bold values indicate statistical significance

### Postoperative pathological assessment

The postoperative pathological characteristics and outcomes of patients in the robotic group were significantly different from those in the laparoscopy group (Table 5). TNM stages II and III were more frequently observed in the robot group than in the laparoscopy group (94.3% vs. 83.2%, *P* < 0.001). There was no significant difference with respect to lymph nodes retrieved between the two groups [13 (7) vs. 13 (6.3), *P* = 0.389]. The prevalence of lymphovascular invasion and CRM involvement also showed no significant difference between the two groups (23.9% vs. 20.0% and 1.3% vs. 0, *P* > 0.05, respectively).

**Table 5:** Postoperative pathological characteristics and outcomes

	Robot (n = 314)	Laparoscopy (n = 220)	*P* value
TNM stage			
I	18 (5.7%)	37 (16.8%)	**< 0.001**
II and III	296 (94.3%)	183 (83.2%)	
Differentiation grade			**0.001**
Well	12 (3.8%)	12 (5.5%)	0.370
Moderate	284 (90.4%)	184 (83.6%)	**0.019**
Poor	17 (5.4%)	12 (5.5%)	0.984
Mucinous	1 (0.3%)	12 (5.5%)	**< 0.001**
Lymph nodes retrieved (no.)	13 (7)	13 (6.3)	0.389
PRM (cm)	10.0 ± 2.9	12.7 ± 3.9	**< 0.001**
DRM (cm)	2.6 ± 0.7	3.1 ± 1.0	**0.022**
Lymphovascular invasion			
No	239 (76.1%)	176 (80.0%)	0.288
Yes	75 (23.9%)	44 (20.0%)	
CRM			
Noninvolved (> 1 mm)	312 (98.7%)	220 (100%)	0.242
Involved (≤ 1 mm)	4 (1.3%)	0	

Data are expressed as the number of patients (percentage). The continuous variables were compared by using the Mann–Whitney U test, and the categorical variables were compared by using the χ^2^ or Fisher’s exact test between the robot and laparoscopy groups

Bold values indicate statistical significance

TNM, tumour node metastasis

## Discussion

In this retrospective comparative cohort study, we found no beneficial survival effect of robotic surgery on patients with rectal cancer compared to those receiving laparoscopic surgery. However, a decreased postoperative complications, operating time, hospital stay and time to 1st gas passing were observed, which revealed a short-term advantages of robotic surgery.

In addition, we found that patients who underwent robotic surgery had a lower tumour location and advanced clinical stage than those who underwent laparoscopic surgery. Operation on these patients especially those with a lower tumour location usually means a more challenging procedure and requires higher skills with surgical techniques than for those without; thus, indicating robotic surgery may have advantages in rectal surgery on more sophisticated cases because of its better visualization and the ability to perform a finer and more dexterous pelvic dissection within a narrow pelvic cavity [15]. Importantly, the time to 1st gas passing and 1st soft diet and the length of hospital stay were significantly shorter in the robot group, indicating that robotic surgery might enhance recovery after surgery. No significant difference was observed with respect to most postoperative short- or long-term complications, while the incidence of urinary retention was significantly decreased in patients who underwent robotic surgery compared to those who underwent laparoscopic surgery, which also indicated the superiority of robotic surgery to laparoscopy for easier identification of the inferior hypogastric plexus.

The postoperative pathological parameters that can measure the quality of rectal surgery are CRM positivity and the number of harvested lymph nodes of the resected specimen; both of which were not significantly different between the robotic and laparoscopic surgical approaches. The CRM involvement rate in this study was 1.3% vs. 0% between the robot and laparoscopy groups, which was comparable with previous studies (0–16%) [5, 7, 12, 16]. In the robot group, there were a total of four patients (4/314) with positive CRMs, and local recurrence occurred in two patients with positive CRMs. However, in the laparoscopy group, there were no cases with positive CRM, and local recurrence occurred in 12 cases (12/224) with negative CRM. A positive CRM did not seem to be translated to local recurrence. That CRM was not a prognostic factor for predicting survival by multivariate analysis, which could support this finding.

Cumulative OS and DFS, the gold-standard prognosticators, indicate long-term oncologic outcomes and reflect the superiority of surgical techniques in cancer resection. Few previous studies have shown the cumulative OS and DFS between the robot and laparoscopy groups. Baek et al. [17] reported that the 3-year OS and DFS were 96.2% and 73.7%, respectively, for patients with stages I-III rectal cancer who underwent robotic surgery in a 1-arm case series study. Pigazzi et al. [18] presented similar 3-year oncologic results of robotic rectal cancer surgery with data from three different centres. Baek et al. [19] also compared the short- and long-term outcomes between robotic and laparoscopic ultralow anterior resection and coloanal anastomosis and reported no difference in local recurrence, 3-year OS, or DFS between the two groups. Park et al. [12] reported that the 5-year OS was 92.8% in robotic surgery and 93.5% in laparoscopic surgical procedures, while the 5-year DFS was 81.9% and 78.7%, respectively. Here, we found that the 5-year OS and DFS were 78.4% and 81.1%, respectively, in patients receiving robotic surgery.

Robotic surgery requires that a surgeon take a long time to learn to adapt to new surgical techniques, such as controlling consoles, manipulating new instruments, and cooperation with the surgical team [20–22]. Our team has adequate experience in robotic surgery, with nearly 200 cases of robotic surgery on rectal cancer per year, which is why our study demonstrated that the operation time and intraoperative bleeding were both significantly less than those of laparoscopy. The high cost of robotic surgery is also a problem, which makes it unable to be widely recommended for patients. Nevertheless, the robotic system is continuously being improved, and more advanced technologies will be developed, such as a novel Senhance^®^ robotic system (TransEnterix Surgical Inc., Morrisville, NC, USA), which has been proven to be feasible and safe in general surgery, gynaecology, and urology [23]. We suppose that the cost of robotic systems will become increasingly acceptable.

## Conclusion

Robotic surgery was not associated with improved survival compared to laparoscopic surgery for rectal cancer, however, robotic surgery is a safe and feasible surgical procedure, especially for some sophisticated cases with lower tumour locations. Further prospective randomized trials are needed to clarify these findings.

## Data Availability

The datasets used and/or analyzed during the current study are available from the corresponding author on reasonable request.

## Abbreviations

TME: Total mesorectal excision
BMI: Body mass index
CEA: Carcinoembryonic antigen
CRT: Chemoradiotherapy
TNM: Tumour node metastasis
OS: Overall survival
DFS: Disease-free survival
CRM: Circumferential resection margin
PRM: Proximal resection margin
DRM: Distal resection margin
HR: Hazard radio
